# The Correlation of Serum Myostatin Levels with Gait Speed in Kidney Transplantation Recipients

**DOI:** 10.3390/ijerph19010465

**Published:** 2022-01-01

**Authors:** Po-Yu Huang, Jen-Pi Tsai, Yen-Cheng Chen, Ming-Che Lee, Bang-Gee Hsu

**Affiliations:** 1 Division of Nephrology, Department of Internal Medicine, Dalin Tzu Chi Hospital, Buddhist Tzu Chi Medical Foundation, Chiayi 62247, Taiwan; poyuhs13628@gmail.com (P.-Y.H.); tsaininimd1491@gmail.com (J.-P.T.); 2 School of Medicine, Tzu Chi University, Hualien 97004, Taiwan; yccmdsurg@gmail.com; 3 Department of Surgery, Hualien Tzu Chi Hospital, Buddhist Tzu Chi Medical Foundation, Hualien 97004, Taiwan; 4 Department of Surgery, Wan Fang Hospital, Taipei Medical University, Taipei City 11696, Taiwan; 5 Division of Nephrology, Hualien Tzu Chi Hospital, Buddhist Tzu Chi Medical Foundation, Hualien 97004, Taiwan

**Keywords:** myostatin, kidney transplant, gait speed, age, body mass index

## Abstract

The primary role of myostatin is to negatively regulate skeletal muscle growth. The gait speed is a noninvasive, reliable parameter that predicts cardiovascular risk and mortality. This study evaluated the relationship between serum myostatin concentrations and gait speeds in patients who had undergone kidney transplantation (KT). A total of 84 KT recipients were evaluated. A speed of less than 1.0 m/s was categorized into the low gait speed group. We measured serum myostatin concentrations with a commercial enzyme-linked immunosorbent assay. KT recipients in the low gait speed group had significantly older age, as well as higher body weight, body mass index (BMI), skeletal muscle index, serum triglyceride levels, glucose levels, and blood urea nitrogen levels, lower estimated glomerular filtration rates and serum myostatin levels, a higher percentage of steroid use, and a lower proportion of mycophenolate mofetil use. Multivariable logistic regression analysis revealed that lower myostatin levels and lower frequency of mycophenolate mofetil use were independently associated with low gait speed. In multivariable stepwise linear regression analysis, myostatin levels were positively correlated with gait speeds, and age and BMI were negatively correlated with gait speeds. In the study, serum myostatin levels were significantly lower in the low gait speed group. Subjects in the low gait speed group also had greater BMI and older age.

## 1. Introduction

Myostatin, a member of the transforming growth factor-β (TGF-β) superfamily, is a negative regulator of skeletal muscle development and growth [1]. It is predominantly produced in skeletal muscles in response to various factors, including inflammatory cytokines, oxidative stress, ammonia, angiotensin II, and glucocorticoids [2]. Knockout of the myostatin gene causes skeletal muscle hypertrophy and hyperplasia [3]. Myostatin up-regulates p21 (a cyclin-dependent kinase inhibitor) and decreases cyclin-dependent kinase 2 (Cdk2) protein levels and activity in myoblasts, inhibiting myoblasts from G1 to S phase of the cell cycle [4]. Myostatin is a key mediator in catabolism within muscle cells and has a significant role in sarcopenia; inhibition of its related signaling pathway can be a therapeutic strategy for management of sarcopenia and possibly its consequences [5]. Treatment of sarcopenia with bimagrumab, a human monoclonal antibody against type II activin receptors, causing them to act as myostatin inhibitors, increased skeletal muscle mass and strength and increased walking speeds [6]. In addition, myostatin expression is also associated with decreased insulin sensitivity [7]. In a rat study, it was found that myostatin inhibition enhanced glucose disposal and glycogen synthesis in skeletal muscle, through increases in levels of GLUT1 and GLUT4 glucose transporters [8].

Chronic kidney disease (CKD) leads to chronic inflammation, an increase in uremic toxins, oxidative stress, and reduced physical activity, all of which are associated with activation of myostatin [2]. Myostatin overexpression in CKD may increase its serum levels. Myostatin concentration is significantly higher in non-dialysis CKD patients than in healthy populations [9]. In detail, indoxyl sulfate upregulates myostatin expression, as noted from animal studies; interleukin (IL)-6 activates the transcription factor, signal transducer, and activator of transcription 3 (Stat3), which then increases myostatin gene expression. Serum myostatin levels are typically higher in patients on hemodialysis than in healthy subjects, but some studies have shown no significant difference between the two groups [9,10,11]. The reasons for the inconsistent results are unknown, but age, gender, nutritional status, and the mode of dialysis can all influence serum myostatin levels.

Myostatin participates in inflammation, remodeling, and fibrosis of vascular walls, as well as the process of atherosclerosis [2,12]. Myostatin mRNA expression increased in the vascular wall of patients with end-stage kidney disease, but without a concomitant increase in myostatin protein expression [13]. Interestingly, in patients undergoing maintenance dialysis, serum myostatin levels were noted to have a positive correlation with the appendicular skeletal mass index and to be negatively associated with the abdominal aortic calcification score [14].

Low serum myostatin levels can be associated with adverse outcomes. In one study, myostatin levels, upon admission to the intensive care unit, were an independent prognostic biomarker for overall survival, with lower myostatin levels (cut-off value, 16.14 ng/mL) being related to poor survival [15]. Additionally, myostatin levels were higher in patients with diabetes than those without diabetes, and low myostatin levels were associated with metabolic syndrome [16].

Walking speed is an important measure of functional status and health, and it has been regarded as the “sixth vital sign” [17]. Sarcopenia is associated with a decline in gait speed and functional dependence [18]. Lower gait speed can predict mortality, especially in the elderly [19]. Low gait speed is a good predictor of morbidity and mortality in patients on hemodialysis [20]. In one systematic review and meta-analysis of cohort studies, each 0.1 m/s decrease in gait speed was associated with an 8% increased risk for cardiovascular diseases and a 14% increased risk for premature mortality [21]. In patients with CKD or on dialysis, the gait speed decreased as CKD progression occurred, as concluded by a systematic review; in this review, while most studies focused primarily on gait speeds as estimation of gait impairments, kidney transplantation patients were not recruited [22]. Furthermore, the relationship among muscle mass, myostatin levels, and gait speeds is not fully known in kidney transplant (KT) recipients. A recent study has found that in KT recipients, patients in the low skeletal muscle index group, compared to those in normal group, had significantly higher serum myostatin concentrations, as well as lower serum brain-derived neurotrophic factor (BDNF) levels; however, there was no significant difference in walking speed, which was determined by the time needed to walk for a distance of 10 m, between the two groups [23]. In this study, we aimed to evaluate the association between gait speed and serum myostatin levels in KT recipients.

## 2. Materials and Methods

### 2.1. Participants

This was a cross-sectional study, and it was conducted in the outpatient department of KT in the medical center in Hualian, Taiwan. Patients who underwent KT for more than 6 months between September 2017 and March 2018 were enrolled in the study. The study was approved by the Research Ethics Committee of Hualien Tzu Chi Hospital, Buddhist Tzu Chi Medical Foundation (IRB104-84-B). These patients were asked to provide written informed consent and were approved by local ethics committee before they were enrolled in the study. The exclusion criteria included active infection within 3 months, acute transplant rejection, decompensated heart failure, malignancy upon enrollment, and unwillingness to provide informed consent. Data regarding baseline characteristics, chronic medication use, and important medical history were collected. History of immunosuppressive drug use, including tacrolimus, mycophenolate mofetil, steroid, rapamycin, and cyclosporine, was obtained through a review of medical records. Hypertension was recognized based on a history of use of antihypertensive drugs, and the diagnosis of diabetes mellitus was established by medical history or use of antidiabetic medications.

### 2.2. Measurement of Blood Pressure, Body Weight, and Height

We measured these participants’ blood pressure using standard mercury sphygmomanometers after a 10 min rest. Body weight was checked with patients wearing light clothing, and height was checked with patients standing barefoot or in their stockings. The body mass index (BMI) was then calculated as weight/height^2^ (kg/m^2^).

### 2.3. Measurement of Skeletal Muscle Index, Handgrip Strength, and Gait Speed

We measured the skeletal muscle mass using a single-frequency bioimpedance device (Tanita BC 706DB, Tanita Corporation, Tokyo, Japan); skeletal muscle index was calculated as skeletal muscle mass/height^2^ (kg/m^2^). Handgrip strength was determined using a Jamar Plus Digital Hand Dynamometer (SI Instruments Pty Ltd., Hilton, Australia) with a precision of 1 kg. Participants were asked to hold the dynamometer in both hands and squeeze as hard as possible, while in an upright standing position, with arms by the side of the trunk, and with elbows flexed at 90°. The measurement on both arms was repeated three times with 1 min rest intervals between each measurement, and we chose the maximum value for further analysis. For gait speed measurement, patients were instructed to walk at their usual pace for six meters on a flat and straight path. The measurements were performed before the initiation of dialysis. A stopwatch was used, and the timing began with a verbal start command (static start). Patients were instructed to maintain their speed without deceleration by the end of the walking course. The gait speed was calculated as the distance traveled (i.e., 6 m) divided by the time taken to cover that distance. The slow gait speed was defined as a gait speed of less than 1 m/s, according to the European Working Group on Sarcopenia in Older People (EWGSOP) criteria. All measurements were performed by the same trained operator.

### 2.4. Biochemical and Myostatin Investigations

Fasting blood samples with a total of about 5 mL were obtained from each participant. Approximately 1 mL of each blood sample was analyzed for hemoglobin level using Sysmex K-1000 (Sysmex American, Mundelein, IL, USA). The other 4 mL were centrifuged at 3000× *g* for 10 min; serum creatinine, blood urea nitrogen, cystatin C, glucose, total cholesterol, triglyceride, and albumin levels were analyzed using an auto-analyzer (Siemens Advia 1800, Siemens Healthcare GmbH, Henkestr, Germany). We used enzyme-linked immunosorbent assays to assess serum intact parathyroid hormone (iPTH) levels (Abcam, Cambridge, MA, USA). The creatinine- and cystatin C-based estimated glomerular filtration rate (eGFR) was determined using the Chronic Kidney Disease Epidemiology Collaboration equation. Serum myostatin levels were checked with a commercial enzyme-linked immunosorbent assay (ELISA) (R&D Systems, Inc., Minneapolis, MN, USA).

### 2.5. Statistical Analysis

Variables with normal distribution were presented as mean ± standard deviation and analyzed using Student′s independent *t*-test or analysis of variance test; variables that were not normally distributed were given as median (interquartile range) and analyzed using the Mann–Whitney U test. Categorical variables were expressed as number and relative proportion [number (%)] and analyzed using chi-squared test. Simple regression or multivariable logistic regression analysis was used to evaluate the factors correlated to low gait speeds. A *p* value of less than 0.05 was considered statistically significant. Statistical analysis was performed by using SPSS 19.0 software (SPSS, Chicago, IL, USA).

## 3. Results

**Figure 1:**
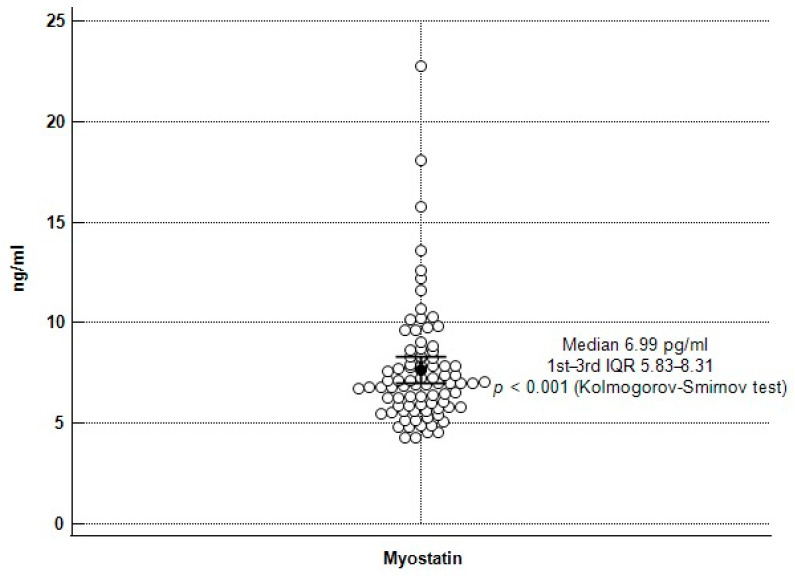
The distribution of serum myostatin concentrations among 84 renal transplant recipients.

A total of 84 KT recipients were enrolled in this study, and the baseline characteristics are shown in Table 1. The distribution of serum myostatin levels is depicted in Figure 1; the variable was not normally distributed, so the logarithm of serum myostatin concentration was used for subsequent linear regression analysis. The mean gait speeds were 1.21 ± 0.15 m/s and 0.86 ± 0.10 m/s in the normal and low gait speed groups, respectively (*p* < 0.001). Patients in the low gait speed group (*n* = 31) were found to be older (*p* = 0.009), to have higher weight (*p* = 0.044) and BMI (*p* = 0.017), and to have a higher skeletal muscle index (*p* = 0.027). Serum triglyceride (*p* = 0.029) and fasting glucose (*p* = 0.007) levels were significantly higher in the low gait speed group. Although there was no statistical difference in the serum creatinine levels between the groups, in the low gait group, there was lower eGFR based on both creatinine (eGFRcre, *p* = 0.047) and cystatin C (eGFRcys, *p* = 0.006), as well as higher cystatin C levels (*p* = 0.015). Mean myostatin levels were 7.61 ng/mL in the normal gait speed group and 6.26 ng/mL in the low gait speed group (*p* < 0.001). The proportion of mycophenolate mofetil use was significantly lower (*p* = 0.001) in the low gait speed group, and the proportion of steroid use was significantly higher (*p* = 0.037) in the low gait speed group.

**Figure 2:**
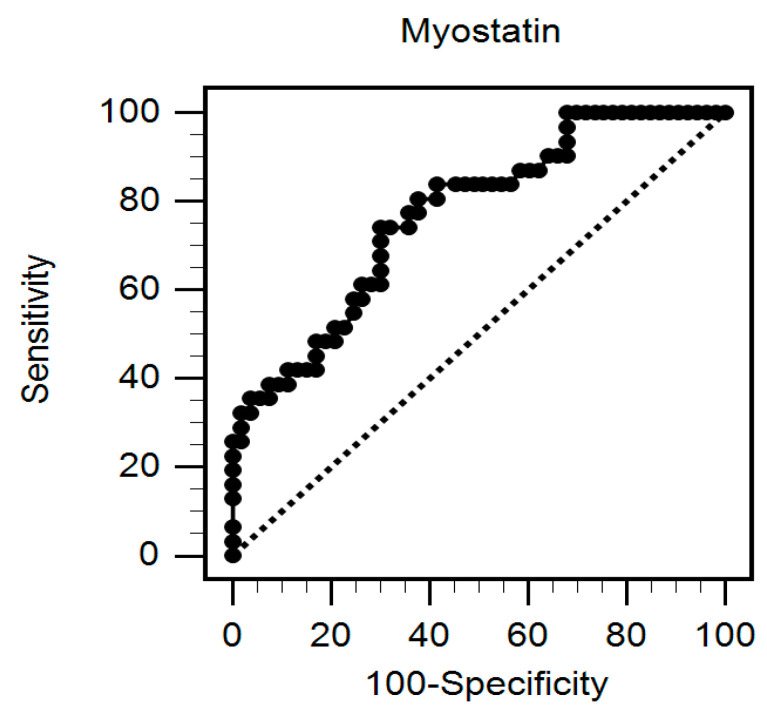
The area under the receiver operating characteristic curve indicates the diagnostic power of serum myostatin values for predicting low gait speed among 84 renal transplant recipients.

Multivariable logistic regression analysis after adjustment for confounding factors revealed that lower myostatin levels (odds ratio [OR] 0.538, 95% confidence interval [CI] 0.327–0.883, *p* = 0.014), as well as a lower usage of mycophenolate mofetil (OR 0.165, 95% CI 0.038–0.720, *p* = 0.017) were independently associated with low gait speed (Table 2). Multivariable stepwise linear regression analysis also showed positive correlation of myostatin levels with gait speeds (β = 0.353, adjusted R^2^ change = 0.245, *p* = 0.001) (Table 3). There was negative correlation of age (β = −0.239, adjusted R^2^ change = 0.033, *p* = 0.016) and body mass index (β = −0.211, adjusted R^2^ change = 0.031, *p* = 0.035) with gait speeds. The area under the receiver operating characteristic curve indicated the diagnostic power of serum myostatin levels for prediction of low gait speed was 0.769 (95% CI: 0.664–0.854, *p* < 0.001) (Figure 2).

**Figure 3:**
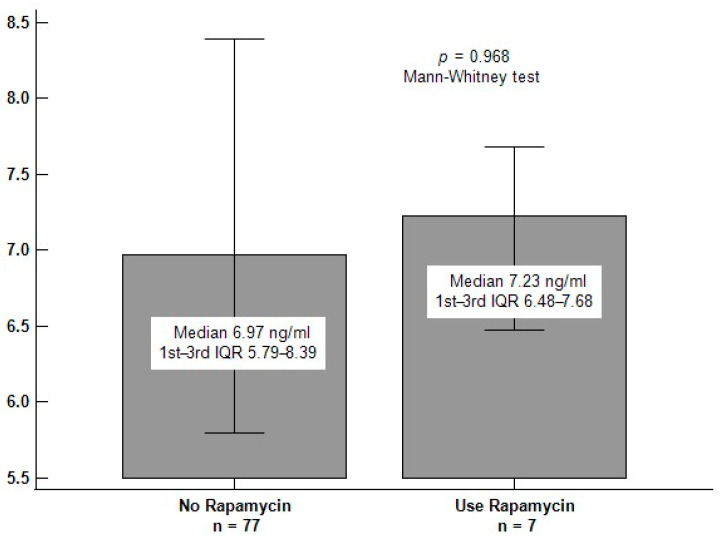
Differences in serum myostatin concentrations between rapamycin users and non-rapamycin users among 84 renal transplant recipients.

There was no significant difference in serum myostatin levels between those who received rapamycin treatment (*n* = 7) and those who did not (*n* = 77) (*p* = 0.968) (Figure 3).

## 4. Discussion

In this study, the most important finding is that lower serum myostatin levels, older age, and higher BMI were independently associated with low gait speeds in KT recipients. Among these factors, lower serum myostatin levels seemed to be the strongest predictor of low gait speeds.

Serum myostatin levels were lower in the low gait speed group. Myostatin, expressed in skeletal muscle, binds to activin receptors type IIB (ActRIIB) on myoblasts, which further transphosphorylate activin type I receptors. Smad 2/3 is phosphorylated and then aggregates with Smad 4. The Smad 2/3 and Smad 4 complex is translocated into the nucleus, blocking the transcription of myogenesis-responsible genes [1,24]. Smad 7 attempts to bind to activin type I receptors and also prevents the formation of the Smad 2/3 and Smad 4 complex. Smad 7 transcription is induced by activin stimulation, which can be regarded as the negative feedback mechanism of myostatin promotor activity and the related signaling pathway [25,26]. Additionally, overexpression of Smad 7 may downregulate endogenous myostatin mRNA levels. As a result, myostatin can auto-regulate its own expression by negative feedback via Smad 7 [25]. The mechanism may explain the decreased levels of serum myostatin in the low gait speed group. In one study that recruited healthy elderly subjects, a positive correlation between serum myostatin levels and gait speed was found [27]. To the best of our knowledge, this is the first study focusing on the association between myostatin levels and gait speeds in patients who have underwent KT.

There was an inverse relationship between BMI and gait speed in our study; in the low gait speed group, the serum triglyceride levels were significantly higher. One possible explanation for higher BMI in the low gait speed group is age-related sarcopenic obesity, which is related to higher fat mass, as well as hypertriglyceridemia [28]. In one cross-sectional study, among the components of metabolic syndrome, low high-density lipoprotein cholesterol in women was significantly associated with lower gait speeds [29]. To sum up, higher BMI may reflect sarcopenic obesity, and dyslipidemia is in part associated with gait speeds.

It is difficult to explain why the skeletal muscle index is slightly higher in patients with lower gait speed. First, a lack of myostatin can lead to excessive muscle growth. Myostatin knockout mice had larger muscle mass but no related increase in maximum tetanic force generation [30,31]. There was increased number of type IIb muscle fibers and a decreased number of type I and IIa fibers, which could contribute to faster fatigue in mice with myostatin depletion. Mitochondrial depletion resulting from lack of myostatin might be associated with easy fatigability and decreased exercise capacity. As a result, lower myostatin levels can increase skeletal muscle mass but lower gait speeds due to faster fatigability. The second explanation is that, as noted above, the higher BMI in the lower gait speed group might indicate sarcopenic obesity. In fact, reference values and cut-offs for skeletal muscle mass or the skeletal muscle index vary widely among different available methods; therefore, it may be impossible to define sarcopenia absolutely by using the skeletal muscle index [32]. Furthermore, obesity may have adverse impacts on skeletal muscle quality, by inducing skeletal muscle inflammation through inflammatory cytokines and chemokines [33]. Despite a higher skeletal muscle index, the skeletal muscle malfunctions, thus causing low gait speeds.

In our study, age is significantly higher in low gait speed group. Aging contributes to sarcopenic obesity with increased fat mass and diminished muscle mass [34]. In one observational study, the proportion of lower gait speeds increased with older ages [35].

It has been shown that in early CKD, plasma myostatin levels are elevated due to decreased renal clearance and increased myostatin production [2]. However, in the lower gait speed group in our study, there was lower eGFR and lower myostatin levels. One possible reason is that myostatin down-regulation is induced by other factors (as mentioned above); another explanation is that myostatin expression at the tissue level, which is not measured in our study, might be more representative. In fact, increased myostatin activation by cytokines, uremic toxins, oxidative stress, and physical inactivity in CKD might not be fully reflected by serum myostatin concentrations [2].

The proportion of mycophenolate mofetil use in patients with low gait speeds is significantly lower, but whether lower frequency of mycophenolate mofetil use is associated with gait speeds is not yet clear. One possible explanation is corticosteroid-induced myopathy. A greater proportion of patients not receiving mycophenolate mofetil treatment (*n* = 30) received steroid therapy (*n* = 28; *p* = 0.047).

Myostatin inhibits the Akt/mammalian target of rapamycin (mTOR) signaling pathway through a decrease in Akt phosphorylation, and the mTOR complex mTORC1 regulates skeletal muscle protein synthesis and muscle hypertrophy [36,37]. As noted from Figure 3, rapamycin use may not have significant impact on serum myostatin levels. Further research is needed to confirm this finding.

Treatment targeting myostatin inhibition may have promising results for patients with sarcopenia or low gait speeds, as mentioned above. Myostatin inhibition therapy may be of benefit, independently of serum myostatin levels, as in our study, the serum myostatin levels are lower in those with lower gait speeds. This warrants further studies for confirmation.

In short, patients in the low gait speed group, when compared to the normal gait speed group, had a significantly more advanced age, greater body weight, greater BMI, higher skeletal muscle index, and lower eGFR, as well as lower myostatin levels.

This study has some limitations. Firstly, the sample size is small and may not be representative of the overall population (especially not of young and middle-aged groups), and this may directly influence the interpretation of the results. Secondly, skeletal muscle mass was measured by using a bioimpedance device, and it did not have ideal reference values for defining sarcopenia. Therefore, we cannot clearly define the association of skeletal muscle mass or index with gait speed values. Thirdly, since this is a cross-sectional study, whether low myostatin levels and low gait speed groups are just in association or have causal relationship is not known. Whether the low gait speed group influences outcomes cannot be demonstrated from the results of this study. Serum myostatin levels may change over time, and the changes might have impacts on skeletal muscle mass, muscle strength, gait speeds, renal functions, or overall survival. Currently, there are limited data to confirm these possible associations. Fourthly, the determination of serum myostatin levels can be easily influenced by conditions that alter myostatin expression, such as age and comorbidities. In addition, currently available ELISA detects only total circulating myostatin, not the precursor protein such as promyostain. Indeed, whether promyostain directly regulates growth of skeletal muscle is not clear. Finally, if we further divide the low gait speed group into “low” and “very low” groups, the association of serum myostatin levels with gait speeds might be even greater; however, the small sample size would make the analysis even more difficult. Different cutoffs for gait speeds might give rise to different results.

## 5. Conclusions

In this study, we can conclude that in patients having undergone KT, serum myostatin levels are positively correlated with gait speed values, and we can predict low gait speeds from myostatin levels. Although gait speed may predict mortality, there is currently no clear, direct evidence to prove that low serum myostatin levels are associated with poor overall survival in KT recipients. Future research can focus on the role of serum myostatin as an independent biomarker for survival in these patients, and an extended follow-up period is mandatory.

## Figures and Tables

**Table 1:** Clinical variables of the 84 renal transplant recipients according to gait speed.

Characteristics	All Patients (*n* = 84)	Normal Gait Speed Group (*n* = 53)	Low Gait Speed Group (*n* = 31)	*p* Value
Age (years)	45.45 ± 10.84	43.11 ± 10.26	49.45 ± 10.79	0.009 *
KT duration (months)	77.00 ± 50.00	80.00 ± 48.00	72.00 ± 54.00	0.515
Height (cm)	161.20 ± 7.60	161.46 ± 7.60	160.74 ± 7.72	0.678
Body weight (kg)	63.38 ± 12.73	61.25 ± 10.01	67.02 ± 15.90	0.044 *
Body mass index (kg/m^2^)	24.36 ± 4.47	23.48 ± 3.46	25.87 ± 5.56	0.017 *
Skeletal muscle index (kg/m^2^)	15.99 ± 2.19	15.59 ± 1.79	16.68 ± 2.63	0.027 *
Left handgrip strength (kg)	25.03 ± 8.54	25.88 ± 8.37	23.57 ± 8.78	0.233
Right handgrip strength (kg)	26.44 ± 9.33	27.59 ± 8.11	24.47 ± 10.97	0.140
Gait speed (m/s)	1.08 ± 0.21	1.21 ± 0.15	0.86 ± 0.10	<0.001 *
Systolic blood pressure (mmHg)	144.14 ± 18.66	146.02 ± 18.40	140.94 ± 18.98	0.231
Diastolic blood pressure (mmHg)	82.27 ± 10.30	83.79 ± 10.21	79.68 ± 10.09	0.077
Hemoglobin (g/dL)	11.91 ± 2.12	12.06 ± 1.97	11.66 ± 2.37	0.417
Total cholesterol (mg/dL)	189.09 ± 45.07	191.64 ± 38.14	184.74 ± 55.39	0.502
Triglyceride (mg/dL)	121.00 (89.25–166.75)	112.00 (86.50–154.00)	148.00 (97.00–206.00)	0.029 *
Fasting glucose (mg/dL)	96.00 (89.25–108.75)	94.00 (88.00–106.00)	100.00 (94.00–136.00)	0.007 *
Blood urea nitrogen (mg/dL)	24.00 (16.00–33.50)	23.00 (15.00–28.00)	26.00 (19.00–48.00)	0.041 *
Creatinine (mg/dL)	1.30 (1.00–1.80)	1.30 (0.95–1.60)	1.40 (1.10–2.10)	0.109
eGFRcre (mL/min/1.73 m^2^)	58.63 ± 26.17	62.96 ± 25.23	51.22 ± 26.49	0.047 *
Cystatin C (mg/L)	1.40 (1.07–1.87)	1.26 (1.04–1.77)	1.61 (1.22–2.20)	0.015 *
eGFRcys (mL/min/1.73 m^2^)	54.00 ± 25.99	59.87 ± 26.02	43.97 ± 23.03	0.006 *
Myostatin (ng/mL)	6.99 (5.82–8.32)	7.61 (6.39–9.73)	6.26 (4.87–7.00)	<0.001 *
Albumin (g/dL)	4.35 ± 0.18	4.37 ± 0.19	4.31 ± 0.18	0.222
iPTH (pg/mL)	91.15 (52.95–146.35)	96.60 (56.08–132.63)	83.60 (48.93–173.65)	0.952
Female, *n* (%)	45 (53.6)	30 (56.6)	15 (48.4)	0.466
Diabetes mellitus, *n* (%)	40 (47.6)	26 (49.1)	14 (45.2)	0.730
Hypertension, *n* (%)	46 (54.8)	28 (52.8)	18 (58.1)	0.642
Living donor, *n* (%)	18 (21.4)	13 (24.5)	5 (16.1)	0.365
Tacrolimus use, *n* (%)	59 (70.2)	34 (64.2)	25 (80.6)	0.111
Mycophenolate mofetil use, *n* (%)	54 (64.3)	41 (77.4)	13 (41.9)	0.001 *
Steroid use, *n* (%)	69 (82.1)	40 (75.5)	29 (93.5)	0.037 *
Rapamycin use, *n* (%)	7 (8.3)	5 (9.4)	2 (6.5)	0.633
Cyclosporine use, *n* (%)	13 (15.5)	9 (17.0)	4 (12.9)	0.618
Statin use, *n* (%)	32 (38.1)	18 (34.0)	14 (45.2)	0.308
Fibrate use, *n* (%)	9 (10.7)	5 (9.4)	4 (12.9)	0.620

Values for continuous variables are given as mean ± standard deviation and tested by Student’s *t*-test; variables that are not normally distributed are given as median and interquartile range and tested using Mann–Whitney U test. KT, kidney transplantation; eGFRcre, estimated glomerular filtration rate from serum creatinine; eGFRcys, estimated glomerular filtration rate from serum cystatin C; iPTH, intact parathyroid hormone. * *p* < 0.05 was considered statistically significant.

**Table 2:** Multivariable logistic regression analysis of the factors correlated to low gait speed among 84 renal transplant recipients.

Variables	Odds Ratio	95% Confidence Interval	*p* Value
Myostatin, 1 ng/mL	0.538	0.327–0.883	0.014 *
Mycophenolate mofetil, used	0.165	0.038–0.720	0.017 *
Age, 1 year	1.043	0.973–1.118	0.234
Body weight, 1 kg	0.966	0.858–1.087	0.563
Body mass index, 1 kg/m^2^	1.068	0.803–1.421	0.650
Skeletal muscle index, 1 kg/m^2^	1.112	0.701–1.764	0.652
Triglyceride, 1 mg/dL	1.006	0.999–1.014	0.091
Fasting glucose, 1 mg/dL	1.009	0.987–1.031	0.439
Blood urea nitrogen, 1 mg/dL	0.993	0.911–1.083	0.879
eGFRcre, 1 mL/min/1.73 m^2^	1.036	0.970–1.107	0.289
Cystatin C, 1 mg/L	1.631	0.256–10.400	0.605
eGFRcys, 1 mL/min/1.73 m^2^	0.977	0.905–1.055	0.553
Albumin	0.180	0.003–9.829	0.401
iPTH	1.008	0.996–1.019	0.181
Steroid, used	0.716	0.081–6.314	0.763

Data analysis was performed using the multivariable logistic regression analysis (adopted factors: mycophenolate mofetil use, steroid use, age, body weight, body mass index, skeletal muscle index, triglyceride, fasting glucose, creatinine, blood urea nitrogen, cystain C, eGFRcre, eGFRcys, myostatin, albumin, and iPTH). eGFRcre, estimated glomerular filtration rate from serum creatinine; eGFRcys, estimated glomerular filtration rate from serum cystatin C; iPTH, intact parathyroid hormone. * *p* < 0.05 was statistically significant.

**Table 3:** Correlation between gait speed values and clinical variables among 84 renal transplant recipients.

Variables	Gait Speed (m/s)				
	Simple Regression		Multivariate Regression		
	*r*	*p* Value	Beta	Adjusted R^2^ Change	*p* Value
Age (years)	−0.348	0.001 *	−0.239	0.033	0.016 *
KT duration (months)	0.030	0.785	—	—	—
Height (cm)	0.035	0.753	—	—	—
Body weight (kg)	−0.298	0.006 *	—	—	—
Body mass index (kg/m^2^)	−0.338	0.002 *	−0.211	0.031	0.035 *
Skeletal muscle index (kg/m^2^)	−0.362	0.001*	—	—	—
Left handgrip strength (kg)	0.025	0.821	—	—	—
Right handgrip strength (kg)	0.132	0.230	—	—	—
Systolic blood pressure (mmHg)	0.170	0.122	—	—	—
Diastolic blood pressure (mmHg)	0.208	0.058	—	—	—
Hemoglobin (g/dL)	0.103	0.353	—	—	—
Total cholesterol (mg/dL)	0.115	0.298	—	—	—
Log-Triglyceride (mg/dL)	−0.218	0.047 *	—	—	—
Log-Glucose (mg/dL)	−0.232	0.032 *	—	—	—
Log-BUN (mg/dL)	−0.213	0.052	—	—	—
Log-Creatinine (mg/dL)	−0.186	0.090	—	—	—
eGFRcre (mL/min/1.73 m^2^)	0.193	0.079	—	—	—
Log-Cystatin C (mg/L)	−0.303	0.005 *	—	—	—
eGFRcys (mL/min/1.73 m^2^)	0.296	0.006 *	—	—	—
Log-Myostatin (ng/mL)	0.504	<0.001 *	0.355	0.245	0.001 *
Albumin (g/dL)	−0.007	0.949	—	—	—
Log-iPTH (pg/mL)	0.065	0.555	—	—	—
Mycophenolate mofetil use	0.246	0.024 *	—	—	—
Steroid use	−0.217	0.047 *	—	—	—
Rapamycin use	−0.023	0.834	—	—	—
Cyclosporine use	0.073	0.507	—	—	—
Statin use	−0.149	0.177	—	—	—
Fibrate use	−0.086	0.439	—	—	—

Data of triglyceride, glucose, BUN, creatinine, cystatin C, and myostatin levels showed skewed distribution and were, therefore, log-transformed before analysis. Data analysis was performed using the univariable linear regression analyses or multivariable stepwise linear regression analysis (adopted factors: mycophenolate mofetil use, steroid use, age, body weight, body mass index, skeletal muscle index, log-triglyceride, log-glucose, log-cystatin C, eGFRcys, and log-myostatin). KT, kidney transplantation; BUN: Blood urea nitrogen; eGFRcre, estimated glomerular filtration rate from serum creatinine; eGFRcys, estimated glomerular filtration rate from serum cystatin C; iPTH, intact parathyroid hormone. * *p* < 0.05 was considered statistically significant.

## Data Availability

The data presented in this study are available on request from the corresponding author.
